# The Comparison of Lichtenstein Procedure with and without Mesh-Fixation for Inguinal Hernia Repair

**DOI:** 10.1155/2016/8041515

**Published:** 2016-04-21

**Authors:** Feyzullah Ersoz, Serdar Culcu, Yigit Duzkoylu, Hasan Bektas, Serkan Sari, Soykan Arikan, Mehmet Mehdi Deniz

**Affiliations:** Istanbul Education and Research Hospital, General Surgery Clinic, Istanbul, Turkey

## Abstract

*Aim.* Although inguinal hernia repair is the most frequently performed surgical procedure in the world, the best repair method has not gained acceptance yet. The ideal repair must be safe, simple, and easy to perform and require minimal dissection which provides enough exploration, maintain patient's comfort in the early stage, and also be cost-effective, reducing operation costs, labor loss, hospital stay, and recurrence.* Materials and Methods.* There were eighty-five patients between the ages of 18 and 75, diagnosed with inguinal hernia in our clinic. Lichtenstein procedure for hernia repair was performed under spinal anesthesia in all patients. Forty-two patients had the standard procedure and, in 43 patients, the polypropylene mesh was used without fixation. All patients were examined and questioned on the 7th day of the operation in terms of pain, scrotal edema, and the presence of seroma and later on in the 6th postoperative month in terms of paresthesia, neuropraxia, and recurrence by a single physician.* Results*. Operative time and pain scores in the nonfixation group were significantly lower, without any increase in rates of recurrence.* Conclusion.* Based on these findings, in Lichtenstein hernia repair method, nonfixation technique can be used safely with better results.

## 1. Introduction

In spite of various techniques being introduced for inguinal hernia repair with new additions, a need for new procedures to decrease recurrence rates and increase patients' life qualities is still demanded. Although results of inguinal hernia operations rely mostly on the operator, a significant difference between the success rates of different techniques has not been observed. Despite the fact that inguinal hernia is a frequent entity in surgical practice, the best repair technique is not clear yet.

Inguinal hernias are seen in 3–8% of the population [1], comprising 80–83% of all hernias. Fifty percent of inguinal hernias are indirect, 25% are direct, and 5% are femoral. Eighty-six percent of all inguinal hernias are found in men, while 84% of femoral hernias are found in women [2, 3]. Indirect inguinal hernia is the most frequent type in both genders. Incidence of strangulation and need for hospitalization increase with aging [4].

The importance of the posterior wall of inguinal canal in etiology and repair has been realised lately. Defects of transverse muscle aponeurosis and fascia have been observed to play an important role in the occurrence of inguinal hernias. The aim of the procedure should be repairing the transverse fascia in a tension-free style.

The high rates of recurrence and testicular complications of conventional anterior repairs have led the surgeons to explore new techniques. The techniques that depend on tissue-supported suturing such as Bassini, Shouldice, Halsted, and McVay have left their places substantially to tension-free repairs with prosthetic meshes, like Lichtenstein, Nyhus, mesh plug, and laparoscopic techniques. In the beginning, meshes were used mainly for incisional hernias, but later they started to be popular also in inguinal repairs, constituting over 80% of all inguinal hernia operations in the United States today. Lichtenstein procedure is the most frequently used method among them.

The use of synthetic meshes for hernia repair was described first by Usher et al. and performed especially for recurrent cases until 1984 [5]. In 1974, Lichtenstein and Shore introduced their technique and reported their results including 1000 patients in 1989 [6]. Hereafter, Lichtenstein procedure with synthetic mesh became accepted as an ideal method for primary inguinal hernias.

The aim of our study is to compare the results of the technique with and without mesh-fixation, in terms of operative time, postoperative pain, complications, and recurrence rates.

## 2. Materials and Methods

Following the approval of the Ethics Committee, 85 patients between the ages of 18 and 75 that had been referred to our clinic between June 2009 and June 2010, diagnosed with inguinal hernia, were evaluated prospectively in our randomized study. Recurrent cases, femoral and bilateral hernias, and patients with the history of collagen tissue diseases and immunosuppressive medications were excluded from the study. All the participants were informed about the study and, following their signed informed consent, the trial was performed in accordance with Helsinki report of clinical trials.

All the patients were admitted to hospital one day before the surgery. The operation area was shaved and cleaned on the operation day. The patients were given 1st-generation cephalosporin at the time of anesthesia induction for prophylaxis. Oral intake was started on the 4th postoperative hour. Uncomplicated cases were discharged on the 1st postoperative day.

All the patients underwent Lichtenstein inguinal hernia repair under spinal anesthesia. Forty-two of the patients were operated on with standard procedure (Group 1), while the synthetic mesh was fixed only around the inguinal cord at the border of the internal ring with one 2-0 prolene suture in 43 patients (Group 2). The rest of the mesh was laid under fascia without any fixations on neither inguinal ligament nor any part of tendon conjoint.

Visual analog scale (VAS) was used to evaluate the pain severity of the patients on the 1st postoperative day. According to this scale, the patients were asked to scale their “current” pain intensity or pain intensity “in the last 24 hours,” and the severity of the pain was scored between 0 and 10 by the patient. All the participants were examined on the 7th postoperative day for seroma formation and scrotal edema and then later on in the 6th month for paresthesia, neuropraxia, and recurrence by a single clinician. Statistical analysis was performed with Number Cruncher Statistical System (NCSS, 2007 Statistical Software, Utah, USA). In addition to the descriptive statistical methods, independent *t*-test was used to compare the groups; chi-square test and odds ratio with the confidence interval of 95% were used for qualitative data. A *p* value under 0.05 was accepted to be statistically significant.

## 3. Results

The mean age and BMI were not found to be statistically significant between the groups, and the *p* values were 0.063 and 0.236, respectively (Table 1).

Region and type of the hernias of the patients were compared and the *p* values were 0.443 and 0.751, respectively, showing no statistical significance (Table 2).

The mean operative time was found to be significantly shorter in group 2 (*p* = 0.001). Duration of hospital stay did not reveal any significance between the groups (*p* = 0.101). Mean VAS score was significantly higher in group 1 (*p* = 0.001) (Table 3).

The differences in the rates of seroma formation, scrotal edema, and recurrence were not found to be statistically significant between the groups, and the *p* values were 0.972, 0.976, and 0.997, respectively. Rates of paresthesia and neuropraxia were not found to be statistically significant between the groups, and the *p* values were 0.625 and 0.543, respectively (Table 4).

## 4. Discussion

Despite the fact that inguinal hernia repair is the most frequent procedure in surgical practice and lots of repair types have been described, efforts to find new techniques have not come to an end, yet. The main factor underlying these searches is to decrease the rates of recurrence. Additionally, applicability, complication rates, hospital stay, labor loss, and overall cost-effectiveness of the techniques have been questioned in the recent years. In these studies, tension-free repair with synthetic mesh has been reported to be superior to other modalities, in both open and laparoscopic surgery [7–10].

The main problem of the conventional hernia repair techniques is the tension on the suture tract, which can be decreased by a relaxation incision but not avoided completely. The primary etiologic factor of the insufficiency of herniorrhaphy is to suture two tissues which do not meet with each other in normal anatomy, in a tense manner, which is also adverse to general surgical principles. Because of the tension, sutures tear the tissues and cause necrosis. Conversely, mesh repairs do not cause tension on the suture tract, enable a repair without changing the normal anatomic configuration, and result in decreased recurrence rates. Additionally, the technique is simple and more effective and causes less pain. Tension-free method also enables performing bilateral hernia repair [11].

For over a century, the success of inguinal hernia repairs is evaluated with their recurrence rates. In a study including 1098 patients by Kark et al., Lichtenstein procedure was reported to have a recurrence rate of 0.1% [12]. Bellone et al. found the same rate following their tension-free repair as 0.8% in 119 patients [13]. McGillicuddy compared Lichtenstein and Shouldice techniques and found the recurrence rates as 0.2% and 1%, respectively [14]. Koninger found recurrences rates of 0.3% following a tension-free repair [15]. Amid et al. studied 4000 patients and followed them up for 5 years and found the recurrence rates as 0.1% in their clinical trials [16–21].

A possible complication of the technique is the nerve compression and vascular damage during the mesh-fixation, which may lead to functional disorders and bleeding. Although it was advised to fix the mesh in laparoscopic repair formerly, studies without mesh-fixation have been performed to avoid these complications and reported favorable results [22].

Similar to these studies, we aimed to perform the standard open technique without mesh-fixation, to decrease complication rates. In the study group, we spread the mesh (of approximately 6 × 10 cm) onto the inguinal region and fixed it only at the border of the internal ring with 2-0 prolene, without fixing it to the tissues. The mean operative time was 32.3 minutes, while it was found to be 49.4 minutes in the control group, which we consider as an important advantage of the technique.

Additionally, postoperative pain was found to be significantly less in the study group, which is one of the most important factors affecting postoperative life quality. Indifference between the groups in terms of hospital stay, postoperative complications, and recurrence rates indicates the safety of the procedure.

## 5. Conclusion

In our study, we performed Lichtenstein procedure with and without mesh-fixation in two groups and compared the results prospectively in terms of patient demographics, postoperative complications, hospital stay, operative time, and effects on life quality. Operative time was found to be statistically shorter, and postoperative pain score was found to be statistically lower in the study group.

Today, new techniques are being explored and introduced frequently in inguinal hernia surgery. Lichtenstein repair, which is accepted to be gold standard in open surgery, may be performed safely and effectively with better results, without mesh-fixation, although further studies with larger control and study groups are necessary for certain results.

## Figures and Tables

**Table 1:** Age and BMI data of the groups.

	Group 1 *n*: 42	Group 2 *n*: 43	*t*	*p*
Age	54.5 ± 12	50.33 ± 12.13	1.88	0.063
BMI	26.14 ± 3.29	25.3 ± 3.21	1.19	0.236

**Table 2:** Distribution of region and type.

		Group 1		Group 2		
Region	Right	24	57.10%	21	48.80%	*χ* ^2^: 0.58
	Left	18	42.90%	22	51.20%	*p* = 0.443

Type	Indirect	23	54.80%	27	62.80%	
	Direct	14	33.30%	12	27.90%	*χ* ^2^: 0.57
	Indirect + direct	5	11.90%	4	9.30%	*p* = 0.751

**Table 3:** Comparison of operative time, hospital stay, and VAS score between the groups.

	Group 1 *n*: 42	Group 2 *n*: 43	*t*	*p*
Operative time (minutes)	49.4 ± 13.17	32.37 ± 7.96	7.24	**0.001**
Hospital stay (days)	1.29 ± 0.46	1.14 ± 0.35	1.66	0.101
VAS	5.88 ± 2.06	3.88 ± 1.78	4.79	**0.001**

**Table 4:** Comparison of seroma, scrotal edema, recurrence, paresthesia, and neuropraxia between the groups.

		Group 1		Group 2			OR 95%
Seroma	**** ****+	4	9.50%	4	9.30%	*χ* ^2^: 0.001	1.02
	**** ****−	38	90.50%	39	90.70%	*p* = 0.972	0.23–4.4

Scrotal edema	**** ****+	3	7.10%	3	7.00%	*χ* ^2^: 0.002	1.02
	**** ****−	39	92.90%	40	93.00%	*p* = 0.976	0.19–5.39

Recurrence	**** ****+	1	2.40%	1	2.30%	*χ* ^2^: 0.0001	1.02
	**** ****−	41	97.60%	42	97.70%	*p* = 0.997	0.06–6.94

Paresthesia	**** ****+	3	7.10%	2	4.70%	*χ* ^2^: 0.024	1.6
	**** ****−	39	92.90%	41	95.30%	*p* = 0.625	0.25–9.95

Neuropraxia	**** ****+	2	4.80%	1	2.30%	*χ* ^2^: 0.370	2.1
	**** ****−	40	95.20%	42	97.70%	*p* = 0.543	0.18–24
